# DDoS attack detection in intelligent transport systems using adaptive neuro-fuzzy inference system

**DOI:** 10.1038/s41598-025-06719-x

**Published:** 2025-07-01

**Authors:** G. Usha, H. Karthikeyan, Kumar Gautam, Nikhil Pachauri

**Affiliations:** 1 Department of Networking and Communications, SRMIST, Kattankulathur, Chennai, 603203 India; 2 Department of Computing Technologies, SRMIST, Kattankulathur, Chennai, 603203 India; 3 Department of Quantum Computing, Quantum Research and Centre of Excellence, New Delhi, India; 4 Quantum Technology, Q-Lab, Vivekananda Institute of Professional Studies- Technical Campus, New Delhi, India; 5 Department of Mechatronics, Manipal Institute of Technology, Manipal Academy of Higher Education, Manipal, 576104 Karnataka India

**Keywords:** Distributed denial of service attack, Intelligent transportation systems, Fuzzy logic, Adaptive neuro-fuzzy inference system, Intrusion detection system, Vehicular network security, Engineering, Electrical and electronic engineering

## Abstract

An intelligent transportation system consists of a variety of applications that analyze and exchange information to reduce traffic, enhance traffic management, lessen the impact on the environment, and boost the advantages of transportation for both business users and the general public. Moreover, Intelligent Transportation Systems is different from the standard vehicular ad hoc network design since it functions in a highly dynamic environment brought on by the quick mobility between the nodes in short connection times. These traits make various threats, weaknesses, and denial-of-service assaults possible. The protection of the intelligent transportation system from attacks and continual maintenance is crucial. In this research work, a Distributed Denial of Service attack detection scheme is proposed to protect the Intelligent Transportation System ecosystem, making use of the Adaptive Neuro-Fuzzy Inference System. By resolving the flaws in the DDoS attack detection methods that are currently in use, the security needs of the Intelligent Transportation Systems ecosystem are taken into account. The learning approach of artificial neural networks and the fuzzy logic model is integrated into the Fuzzy System. Based on the experimental results, the proposed model achieved 94.3% accuracy, outperforming traditional classifiers such as Support Vector Machine, Random Forest, Extreme Gradient Boosting, and Convolutional Neural Network. The system demonstrated low false positive rates and high detection reliability, ensuring suitability for real-world Intelligent Transportation Systems security. The proposed scheme attained better results in terms of accuracy, precision, recall, and F1 score.

**Supplementary Information:**

The online version contains supplementary material available at 10.1038/s41598-025-06719-x.

## Introduction

According to projections, the market for intelligent transportation systems would increase from $22.91 billion in 2021 to $42.80 billion in 2028, with a CAGR of 9.34% during that period^1^. The industry for intelligent transportation systems (ITS) is expanding as a result of a variety of factors, including the rising demand for traffic control solutions, vehicles, better safety and surveillance provided by contemporary cameras, License Plate Recognition (LPR) technology, and the quickening development of smart cities. Positive market prospects are produced by Intelligent Transportation Systems’ provision of traffic management systems that improve mobility, traffic flow, and road safety. The COVID-19 epidemic has impeded the sales and installation of new Intelligent Transportation Systems in 2020. However, the Intelligent Transportation Systems sector experienced major growth in the middle of 2021 as a result of increased funding from various governments for infrastructure development projects aimed at reviving the post-pandemic economy.

The number of vehicles on the road is increasing, the infrastructure is ageing, and there is a shortage of traffic data management, among other things, which are all expected to support the expansion of the Intelligent Transportation Systems market. Therefore, there is a growing need for better traffic management technology to control traffic flow on highways and in city corridors. Many initiatives have been made by both public and private entities to implement smart technologies, showing how technology will influence the development of the intelligent transportation system market. The Intelligent Transportation Systems solution includes a Traffic Management Center (TMC), which can handle real-time data and help cars discover alternate routes to minimize traffic issues.

The TMC solution consists of several systems and components, including sensors, vehicle probes, navigation systems, and video surveillance systems. Dynamic signboards, the internet, or mobile telephony are used to analyze, process, and transmit to the user the results of the data acquired by devices. The use of Intelligent Transportation Systems is anticipated to increase over the projected period, notably due to developments in sensing and communications technology. But one of the key drivers of the Intelligent Transportation Systems industry’s expansion is the growing preference for the digitalization of numerous features for the implementation of intelligent transportation system solutions.

One of the main factors propelling the market’s growth is the adoption and widespread use of Intelligent Transportation Systems to lower traffic accidents and improve road safety. In addition, it is anticipated that over the coming few years, demand for vehicle-to-infrastructure (V2I) and vehicle-to-vehicle (V2V) communication will continue to grow^2,3^. The continued advancements in the transportation sector, such as blind-spot detection and electronic toll collection, have heightened the need for an effective transportation system that has the potential to enhance road networks and reframe the anticipations and possibilities of self-sustaining transportation and traffic management. Over 30% of the market in 2022 was occupied by the Advanced Traffic Management System (ATMS) component.

DoS attacks are carried out by dispersing the most data packets possible to interfere with network functioning^4–7^. When an attack is launched from a single source, it is much easier to identify and mitigate than when it is launched with a large number of data packets, which can severely harm the system. Attackers often use a large number of compromised nodes to conduct DDoS assaults to avoid being easily detected. On the other hand, despite taking into account low-rate DDoS attacks, attackers use them to complicate their detection and mitigation as well. Sincere attention is being paid in this work to both high- and low-rate DDoS attacks^8,9^.

This research work, which primarily addresses DDoS attacks, depends on the safety of the people participating in the Intelligent Transportation Systems ecosystem as well as the safety of Intelligent Transportation Systems nodes. The proposed scheme will help monitor the ITS environment for malicious activity, identifying traits of distributed denial of service (DDoS) assaults and DoS attacks. Possibilities are made to lessen dangers and chances of deaths by minimising assailants’ malevolent acts to safeguard the lives of pedestrians and vehicle users.

Despite the growing body of work on intrusion detection in vehicular networks, most existing solutions suffer from one or more of the following limitations: high false positive rates, lack of adaptability to dynamic traffic conditions, dependency on complex optimization techniques, or computational inefficiency. Deep learning models often demand significant processing power, making real-time deployment in intelligent transportation systems impractical. Traditional machine learning models lack the flexibility to generalize to unseen attack patterns in fast-changing vehicular environments. This creates a significant research gap in developing a lightweight, interpretable, and adaptive intrusion detection framework that can operate effectively in real-time ITS scenarios. The proposed adaptive neuro-fuzzy inference system-based approach addresses this gap by integrating fuzzy decision rules with neural learning, offering a novel and efficient alternative tailored to the dynamic nature of ITS security. The proposed approach effectively reduces false alarms and enhances detection accuracy, making it a robust solution for real-time ITS cybersecurity.

The research’s main contributions are listed below as follows.

1. ANFIS-based DDoS Detection Framework: We propose a novel detection framework leveraging Adaptive Neuro-Fuzzy Inference System (ANFIS), which combines the adaptive learning of neural networks with the decision-making capabilities of fuzzy logic. This integration ensures better handling of dynamic vehicular traffic in real-time ITS environments.
2. Entropy and Information Gain-based Feature Selection: An effective feature selection strategy using entropy and Information Gain is employed to identify the most relevant features from the UNSW-NB15 and CICDDoS2019 datasets. This enhances classification performance while reducing computational complexity.
3. Dual-Dataset Evaluation and Simulation: The proposed model is evaluated using two widely accepted intrusion detection datasets (UNSW-NB15 and CICDDoS2019). Additionally, a simulated DDoS environment is created using the LOCUST tool to validate real-time detection capabilities.
4. Comprehensive Comparative Analysis: Extensive experiments demonstrate that the proposed ANFIS model outperforms traditional machine learning classifiers (SVM, RF, XGBoost, CNN) across all key performance metrics—accuracy, precision, recall, F1-score—while maintaining a low false positive rate and computational efficiency.

The organization of this paper is divided into the following sections: In Section “Related works”, security risks, threats, and countermeasures for Internet of Things security are discussed. In Section “Methodology”, a literature survey of recently proposed DDoS attack detection methods is covered. In Section “Results”, the proposed Hybrid Deep CNN model-based DDoS flooding attack detection framework is discussed. In Section “Discussion”, the experimental results are presented. In Section “Conclusion”, the conclusion and future work are mentioned.

## Related works

In the field of ITS and Vehicular networks security, abundant research works have been carried out, mostly on DDoS attacks detection^10^ and mitigation. Recently carried out research works are accumulated in this section, and Table 1 provides a summary of recent research works.

To quickly identify and remove suspicious sensors that generated a distributed denial of service (DDoS) attack in the IVN, a joint K-means clustering and software-defined networking removal framework is suggested^11^. This framework uses software-defined networking and machine learning. The proposed JKS is integrated into the current network architecture and has the capacity to quickly identify and stop a DDoS attack on the IVN. The SDN controller instructs the necessary switches to deny the DDoS attack when the K-means algorithm has recognized it. Once the attack is over or the traffic flow has returned to normal, the stopped flows can be forwarded to the network once more. Once the attack is over or the traffic flow is restored to normal, the stopped flows can be redirected to the network.

The architecture of a MEC-enabled SDVN system is proposed and includes edge DDoS attack mitigation and computation offloading^12^. When allocating resources, the architecture assesses the trustworthiness of the cars and takes it into account. It also distributes computing workloads among MEC servers to balance the demands placed on those servers and the available computing resources. The goal of MEC-enabled SDVN’s resource allocation and mitigation is to reduce energy consumption and latency across all computing offloading and transferring tasks. To find a solution to the stated optimization problem, an integrated algorithm called GCDRL is suggested. Amongst those, the convolutional neural network (CNN) and the generalised neural network (GNN) are utilised to extract, respectively, vehicle spatial data and structure features of edge nodes. In addition, the temporal features of the full state space are extracted using long short-term memory (LSTM). To compare the suggested algorithm’s throughput, latency, and energy usage to those of alternative approaches, experiments are carried out to assess its performance. According to the results of the experiments, the GCDRL approach successfully lessens the negative impacts caused by edge DDoS attacks.

A method for dealing with the security and authentication problems that affect vehicles in VANET is presented^13^. This approach makes a substantial contribution to the intelligent transport communication network by authenticating cars in the VANET and detecting various cyberattacks like DDoS. Identity-based encryption is used in this strategy to manage vehicle access, and deep learning-based approaches are used to filter malicious traffic. A cutting-edge deep learning approach detects fraudulent packets with an accuracy of 99.72% and is IND-sID-CCA secure for this identity-based encryption method. The findings highlight the applicability of the suggested strategy for VANETs in 6G communication systems.

A feature adaptation reinforcement learning strategy called FAST is suggested using reinforcement learning, and it is based on the space-time flow regularities in IoV for DDoS mitigation^14^. A feature adaptation reinforcement learning strategy called FAST is suggested using reinforcement learning, and it is based on the space-time flow regularities in IoV for DDoS mitigation. Furthermore, by integrating Q-learning with DDQN, FAST may choose features and detect DDoS attacks in an environment-aware manner. The Shenzhen taxicab dataset is used in tests to assess FAST’s performance. Two distinct DDoS simulation tools, namely “ddosflowgen” and “hping3”, are utilised to simulate and deploy DDoS attacks into Shenzhen taxicabs.

In IoV, a Real-Time Edge Detection Scheme is presented for Sybil DDoS. The traffic distribution is measured using the entropy theory, and a Fast Quartile Deviation Check (FQDC) algorithm is then developed to detect and identify the attack^15^. The computation is further optimised with various helpful techniques, like the optimised sliding window and the incremental calculation of entropy values, to make it feasible for the IoV environment due to the calculation limitation in IoV scenarios. A temporal index is then suggested. The effectiveness of response speed and omission rate is measured using the Temporal False Omission Rate (TFOR). With an average warning latency of 4.9193 s and an average TFOR of 1.6024%, all Sybil DDoS attacks presented in the F2MD datasets are successfully identified during evaluation.

To enhance cloud vehicle service security, an intelligent application protection methodology is developed for smart car services in a Vehicle to Cloud (V2C) environment^16^. Anomalous activity detection is performed via image-based system resource monitoring, making use of AI (ISRM-AI). While using V2C cloud services, the ISRM-AI creates pictures of system data like CPU, network, and memory. Additionally, the method uses a convolutional neural network (CNN) for evaluating the status to identify any anomalous service behaviour. A service environment is built to evaluate the effectiveness of the suggested technique. Furthermore, using actual attack data, the suggested mechanism’s capacity to identify DDoS attacks is simulated. The suggested approach strengthens the security of the V2C environment to ensure the dependability of a smart service. Based on the simulation results, the host system’s GPU utilization is obtained as 0.2% with a 7.36% detection error rate.

Trust trust-based DDoS attack detection mechanism is proposed for the VANET environment^17^. Frequency value statistics, trust hypothesis statistics, residual energy, trust policy, and data factor are considered as the main trust components in trust evaluation, and a trust evaluation matrix is created based on trust elements. The proposed trust mechanism was created in a way to improve security by keeping outsiders out of the network. Based on the experimental results, the proposed trust mechanism attained a 95.8% detection rate, which is observed as better results than the prevailing techniques like Firecol and AODV.

Based on big data technologies, a distributed DDoS network intrusion detection system is suggested for the VANET environment^18^. It is comprised of two modules, which are real-time network traffic collection and network traffic detection. It is developed making use of Spark and HDFS, which are used for data processing and storing massive suspicious attacks, respectively. The micro-batch data processing methodology is employed in the network collection module to enhance the performance level of traffic feature gathering. The classification technique based on Random Forest (RF) is employed by the traffic detection module. Using the NSL-KDD and UNSW-NB15 datasets, respectively, the experimental findings demonstrate that the suggested detection algorithm achieved accuracy rates of 99.95% and 98.75%, as well as false alarm rates (FAR) of 0.05% and 1.08%.

To detect and isolate DDoS attacks in VANET, reCAPTCHA evaluation is done by a density-based attack analyzer during the attack scenario. Similar to botnet zombies, the reCAPTCHA controller mechanism stops automated attacks. Every rule within the rule metrics indicates an instance of traffic being filtered to block a certain IP address or port. Each rule’s frequency and entropy value are calculated for all incoming packets during every detection window. The difference between the stored traffic profiles and the recent ones indicates a high likelihood of assault. The belief scores are determined by deviating each detection’s present score from its past score, and they are updated at the conclusion. Additionally, it improves the Packet Delivery Ratio (PDR) and DR while minimizing latency and energy consumption as evaluated by parameters AL and EC, accordingly. The DR controller technique is proposed^19^. Network performance for the suggested reCAPTCHA Controller technique is 94.7% according to the experimentation results.

Based on software-defined networking (SDN), a platform is created to effectively identify and quickly respond to DDoS attacks in VNs^20^. The suggested platform includes a flow feature extraction approach that uses multidimensional information in addition to a trigger mechanism based on the OpenFlow protocol message (i.e., PACKET IN message) for an untimely response. Based on the OpenFlow flow table feature and the flow table entry entropy feature, an efficient global network flow table feature values are also established. A trained SVM is utilized for identifying all flow table entries. Based on experimentation, various kernel functions of SVM are analysed, and the linear kernel function attained a 97.68% Detection Rate (DR).

In recent years, artificial intelligence models integrated with metaheuristic optimization techniques have emerged as effective solutions for enhancing intrusion detection systems (IDS) across IoT, IIoT, and vehicular environments. Kocherla et al.^21^ developed a stacked recurrent neural network (RNN) architecture combined with bio-inspired optimization to improve detection performance in Industrial Control Systems. Similarly, Alzubi et al.^4^ proposed an IoT intrusion detection system using the Salp Swarm Algorithm (SSA) for tuning an artificial neural network (ANN), which improved convergence and classification accuracy.

Hybrid machine learning models have also been explored, particularly using XGBoost as the core classifier. XGBoost tuned with Hybridized Sine Cosine Algorithm (SCA) metaheuristics demonstrated notable improvements in Healthcare 4.0 IoT scenarios, where tuning allowed the model to adapt dynamically to complex patterns. Moreover, the Firefly Algorithm and its improved variants have been employed to optimize XGBoost’s hyperparameters, yielding high detection rates with reduced false positives in network security applications. These optimization strategies enable enhanced learning, but they also introduce computational overhead and require careful parameter selection.

In another stream of research, K-means clustering has been combined with metaheuristic algorithms such as Genetic Algorithm and Firefly Algorithm for improved anomaly grouping and feature refinement in IDS. These hybrid methods offer better interpretability but often require iterative adjustments and computational resources, which may limit real-time applicability in highly dynamic environments such as ITS.

While these approaches exhibit strong detection capabilities, the proposed ANFIS-based model differs by offering a lightweight and interpretable hybrid framework that combines fuzzy logic with neural network learning. This eliminates the dependency on heavy optimization routines and ensures real-time adaptability, making it better suited for the ITS ecosystem.

Intrusion detection systems (IDS) leveraging artificial intelligence have gained significant traction in cybersecurity. Several studies have integrated machine learning with metaheuristic optimization techniques to enhance detection accuracy and reduce false positives. For example, K-means clustering combined with metaheuristic methods, such as Genetic Algorithms (GA) and Firefly Algorithm, has been proposed to optimize anomaly detection in IoT networks^21^. Similarly, XGBoost-based IDS models have been fine-tuned using Hybridized SCA Metaheuristics, yielding significant improvements in intrusion detection within Healthcare 4.0 IoT systems.

Beyond traditional classifiers, deep learning models such as CNNs and LSTMs have also been explored for network security. Recent works have implemented hybrid CNN-GNN frameworks to extract spatial and structural network traffic features for anomaly detection in ITS^22^. However, these models often suffer from high computational overhead, making them less practical for real-time intrusion detection in ITS environments. In contrast, the proposed ANFIS-based approach in this study leverages fuzzy logic and neural networks to achieve a balance between accuracy and computational efficiency, making it well-suited for the dynamic nature of vehicular networks.

Furthermore, studies have explored the application of metaheuristic optimization in IDS across various domains such as IoT, IIoT, and cloud security. The hybridization of optimization techniques such as the Firefly Algorithm and Improved SCA has demonstrated enhanced accuracy in IDS models^4^. Given the adaptability of these approaches, future research may explore their potential integration with ANFIS for further improvement in ITS cybersecurity.

In SCADA-based networks, a comparative analysis was conducted to evaluate two countermeasure techniques using sniffers for detecting DDoS attacks^23^. The study demonstrated the role of lightweight monitoring tools in identifying attack patterns effectively within a structured industrial environment. While SCADA systems are relatively static, Intelligent Transportation Systems (ITS) operate under dynamic, real-time conditions. Therefore, detection models designed for ITS, such as our proposed ANFIS-based approach, must be adaptable to rapidly changing traffic patterns and mobile network elements.

In the IoT domain, a novel intrusion detection framework was proposed using Decisive Red Fox Optimization and a back-propagated radial basis function neural network^24^. This hybrid approach demonstrated improved detection accuracy by optimizing the neural network structure for enhanced learning. Such optimization strategies are powerful but may introduce computational overhead. In contrast, our ANFIS-based approach offers a lightweight and interpretable solution more suitable for time-sensitive ITS environments.

Thiruppathy Kesavan et al.^25^ proposed an ANFIS-PSO hybrid model to enhance rule tuning in VANETs, addressing scalability and precision limitations in fuzzy inference. This highlights the potential of optimization algorithms to enhance adaptability in evolving environments.

In a recent approach targeting smart city cybersecurity, a hybrid framework was proposed using Conjugate Self-Organizing Migration (CSOM) and Reconciliate Multi-agent Markov Learning (RMML) under a cyborg intelligence paradigm^26^. This multi-agent strategy enables distributed detection and response, making it suitable for large-scale dynamic environments. While such models offer high adaptability and scalability, they are complex to train and deploy. In contrast, the proposed ANFIS-based framework provides a more lightweight and interpretable solution, with scope for future integration of multi-agent learning in ITS contexts.

A comprehensive study of modern optimization techniques and their applicability to engineering challenges was recently presented, covering methods such as swarm intelligence, genetic algorithms, and evolutionary computation^27^. These techniques offer strong potential for enhancing feature selection and model training in security-sensitive environments such as ITS. In our work, while ANFIS does not employ such optimization directly, future research may consider integrating such methods to fine-tune fuzzy rules dynamically^28,29^.

For real-time intrusion detection in SCADA/IoT settings, Bhukya, Raghuram, et al.^30^ introduced the SPARK and SAD frameworks, which demonstrated superior computational efficiency compared to traditional deep learning approaches. Their architecture offers insights applicable to latency-sensitive ITS systems.

Selvarajan, Shitharth, et al.^31^ employed adversarial learning techniques to enhance the robustness of cyber-physical systems against evolving threats. Such techniques could be incorporated into future iterations of the ANFIS-based framework to improve resistance to adversarial perturbations.

**Table 1:** Summary of recent works.

Research Work	Features	Algorithms of classification	Detection method
Huang et al. (2022)^20^	Machine learning, SDN controller	K-means algorithm	Joint K-means clustering
Deng et al. (2022)^19^	Optimization problem, CNN, GNN, LSTM	GCDRL	MEC-enabled SDVN system
Zhou et al. (2022)^18^	Identity-based encryption, 6G	Deep learning-based approaches	DL based
Li et al. (2022)^17^	Ddosflowgen, hping3, Shenzhen taxicabs	Reinforcement learning	FAST
Li et al. (2021)^16^	Temporal index, F2MD dataset	FQDC, entropy theory	Real-Time Edge Detection Scheme
Kim et al. (2020)^15^	CPU, network, and memory	CNN	ISRM-AI
Poongodi et al. (2019)^14^	Frequency value statistics, trust hypothesis statistics, residual energy, trust policy	Trust mechanism	Trust based
Gao et al. (2019)^13^	Spark and HDFS, NSL-KDD, and UNSW-NB15 datasets	RF	Big data technologies based
Poongodi et al. (2019)^12^	Rule frequency and entropy value, belief score	reCAPTCHA controller technique	reCAPTCHA controller technique
Guo et al. (2018)^11^	Trigger mechanism, multidimensional information	SVM	SDN based

## Methodology

**Fig. 1:**
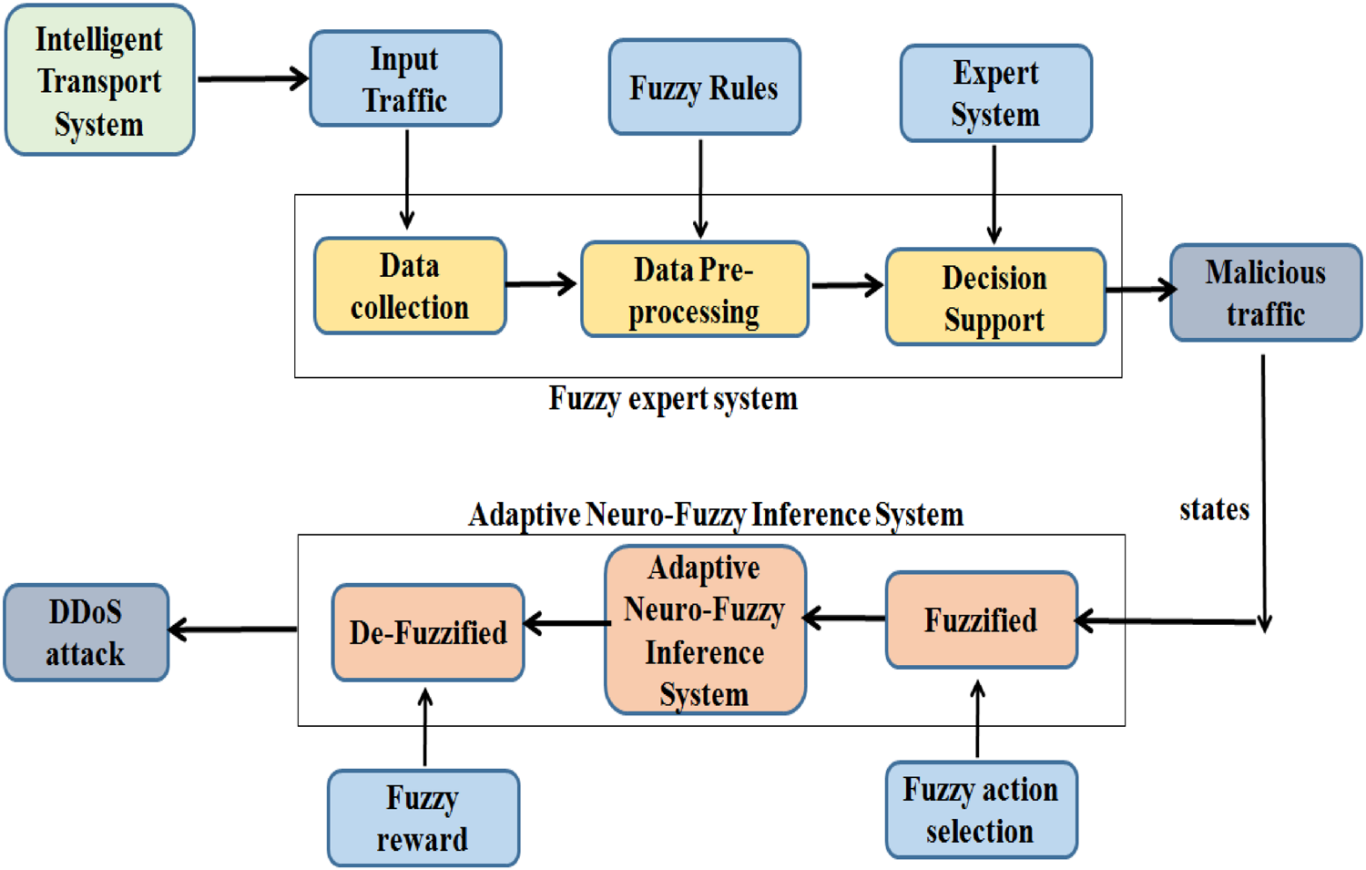
Proposed DDoS detection scheme.

This section details the components of the proposed ANFIS-based DDoS detection scheme for the ITS environment in Fig. 1. Figure 3 depicts the complete proposed scheme, which includes two major parts which are Fuzzy Expert System and the Adaptive Neuro-Fuzzy Inference System.

### Fuzzy expert system

#### Data collection

The ITS ecosystem is heavily dependent on data, and many ITS applications generate, analyse, store, and communicate that data. Data requirements can be broken down into multiple categories, including background information, configurations, data collected, daily functional inputs, real-time data obtained from ITS devices as well as ITS applications, and downstream processing data. Two distinct datasets are utilized for experimental purposes, which are the UNSW-NB15 dataset and the CICDDoS2019 dataset.

#### Data pre-processing

Pre-processing is primarily utilized to arrange and clean unprocessed data to make sure it is suitable for building the model. Pre-processing is applied to input traffic data collected from the ITS ecosystem. After pre-processing, features like utilization, packet rate, byte rate, packet delay, and packet size are considered for selecting the appropriate features from the DDoS datasets. The Information Gain (IG) algorithm is used for feature selection. The IG algorithm relies on entropy, and entropy is computed for categorizing an appropriate feature. If E=(E, P) is discrete probability space in which E = {E_1_,E_2_,….E_n_} is defined as a finite set of chosen features. Entropy is formulated as follows.

1 $$\:\text{E}\text{n}\text{t}\text{r}\text{o}\text{p}\text{y}\:\left(\text{E}\right)=-\sum\:_{i}{p}_{i}{log}_{2\:}{(p}_{i}) \, \,\,\,\, \, i= 1,2…n$$

In Eq. (1), *p*_*i*_ represents the probability of class *i* occurring within the dataset, and n denotes the total number of distinct classes. Entropy H(E) quantifies the uncertainty or impurity in the data distribution.

Once entropy is computed, then IG value computation is done based on the previously computed entropy value, and it is formulated as follows.

2 $$\:IG=Entropy\:\left(E\right)-\sum\:\frac{{E}_{K}}{n}Entropy\:\left({E}_{K}\right).$$

In Eq. (2), Information Gain IG(E) is computed as the difference between the entropy of the entire dataset H(E) and the weighted sum of entropies of each subset H_k_​(E). Here, n_k_​ represents the number of instances in subset k, and n is the total number of instances in the dataset. The term $\:\frac{{n}_{k}}{n}$​​ Acts as the weight for each subset’s entropy.

#### Decision support

The foundation of fuzzy logic is a set of multiple-valued rules, where the variables’ true values fall between 0 and 1. It will be regarded as a subset of AI and used mostly in the decision-making process. In a real-world setting, there may be some complex circumstances where it is impossible to determine if something is true or false. Fuzzy logic simplifies decision-making in this scenario.

In a Boolean system, the truth values are shown with absolute truth values of 1 and 0; however, in a fuzzy logic system, no logic is applied to the fixed truth and false values, and instead, it is made up of in-between values that are utilized to display partially true and false values. Membership functions are also used in the fuzzy logic system to transfer values between 0 and 1 to graph representations.

All available data is taken into account by the fuzzy logic algorithm while addressing problems. From the supplied input, the best possible decision is further determined. FL resembles the decision-making procedure of a human being, which emphasizes every alternative between the T value and the F value. The idea of fuzzy logic dates back to the 1920s, but Lotfi Zadeh coined the phrase in 1965 after observing how traditional computer logic was utilized to manipulate data.

One of the crucial components of the fuzzy system is the section responsible for fuzzy rule generation. The fuzzy rule basis of the suggested scheme was produced to identify the level of attack using a straightforward technique. The fuzzy rules were created using professional knowledge of DDOS attack detection, collected range values (Low, Medium, High), and extensive research into the correlation between the most important aspects and DDOS intrusion detection.

**Fig. 2:**
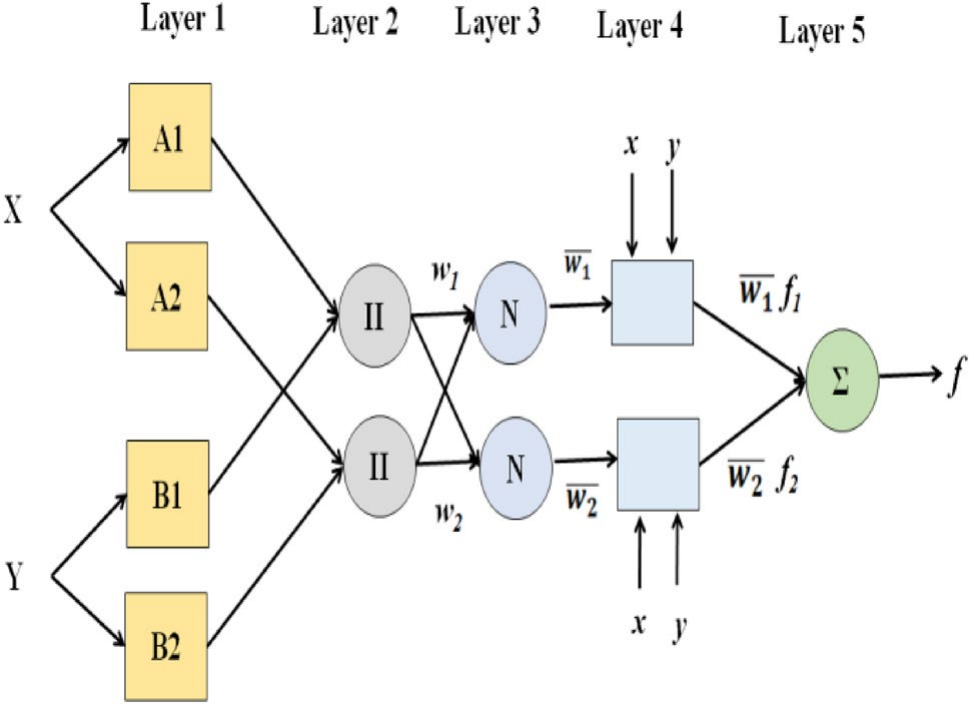
ANFIS architecture adapted for the proposed solution.

### Adaptive neuro-fuzzy inference systems

Adaptive Neuro-Fuzzy Inference Systems (ANFIS), created by J.S. Roger Jang in 1993. ANFIS is often referred to as the standard estimator or Takagi-Sugeno Fuzzy System. In this method, the fuzzy logic model and the learning method for artificial neural networks (ANN) are combined. To establish the ideal distribution of membership functions, ANFIS assesses and simulates the mapping relationship between the input and output data over a hybrid system.

Selection of ANFIS for ITS Intrusion Detection:

In designing an intrusion detection system for Intelligent Transportation Systems (ITS), it was critical to select a classification model that could efficiently handle the unique challenges posed by vehicular networks. These include high mobility, real-time processing demands, unpredictable traffic flow, and heterogeneous network conditions. Several machine learning classifiers were considered, including Support Vector Machines (SVM), Decision Trees (DT), Random Forest (RF), XGBoost, and Deep Neural Networks (DNN).

We conducted an initial evaluation of these classifiers using sample subsets of CICDDoS2019 and UNSW-NB15 datasets. The evaluation was based on the following criteria:

Detection Accuracy: The ability to correctly classify normal and attack traffic.

Computational Efficiency: The feasibility of deployment in real-time ITS environments.

Scalability: The ability to handle large, high-speed network data.

Adaptability: The ability to handle evolving attack patterns in ITS networks.

Key Observations from Initial Evaluations.

Traditional classifiers (SVM, Decision Trees, and Random Forest) showed moderate accuracy but struggled with dynamic, evolving attack types, leading to higher false positives. Gradient boosting models (e.g., XGBoost) performed well in terms of accuracy but required significant hyperparameter tuning and had longer processing times, making them less suitable for real-time deployment. Deep learning models (CNN, LSTM, and DNN) achieved high accuracy but were computationally expensive, requiring large training datasets and specialized hardware, which may not be feasible in real-world ITS deployments.

Several classification models were evaluated, including Support Vector Machine (SVM), Decision Tree (DT), Random Forest (RF), Extreme Gradient Boosting (XGBoost), and Convolutional Neural Network (CNN), using benchmark datasets (UNSW-NB15 and CICDDoS2019). While SVM and DT showed moderate accuracy, they lacked adaptability to dynamic traffic patterns. RF improved stability but was computationally heavier. XGBoost achieved high accuracy but required intensive tuning, and CNN was computationally expensive, limiting real-time applicability.

In contrast, the Adaptive Neuro-Fuzzy Inference System (ANFIS) offered a balanced solution—combining fuzzy logic for interpretability with neural learning for adaptability. It demonstrated real-time efficiency, reduced false positives, and required lower computational resources. These factors made ANFIS well-suited for the dynamic and resource-constrained intelligent transportation system environment.

After careful consideration, ANFIS (Adaptive Neuro-Fuzzy Inference System) was chosen because it provides an optimal balance between accuracy, interpretability, and adaptability:

Hybrid Intelligence: ANFIS merges fuzzy logic (for handling uncertainty in vehicular traffic) with neural networks (for learning from evolving attack patterns), making it well-suited for the dynamic ITS environment.

Reduced False Positives: The combination of rule-based decision-making (fuzzy logic) and adaptive learning (neural networks) resulted in lower false positive rates compared to SVM and XGBoost.

Lightweight and Efficient: Unlike deep learning models, ANFIS does not require massive computational power, making it more practical for real-time intrusion detection.

Self-Adaptive Model: ANFIS can dynamically adjust detection rules as traffic conditions change, an essential feature for high-speed vehicular networks.

Performance Evaluation Summary.

Final model testing using the full CICDDoS2019 and UNSW-NB15 datasets confirmed that ANFIS outperformed traditional classifiers in terms of both accuracy and real-time efficiency, making it the most suitable choice for ITS security applications.

ANFIS is comprised with five layers which are fuzzification, implication, normalization, defuzzification, and summation. The node function defines a number of nodes that make up these layers. The same thing can be done with nodes in each layer. The nodes’ adjustable parameters have a major impact on the network output. The nodes’ adjustable parameters have a major impact on the network output. With the purpose of reducing error, the network learning rules change these parameters. Figure 2 displays the ANFIS architecture, which has two inputs as well as one output. Two if-then rules based on an inference system of the Takagi-Sugeno type are taken into consideration in order to make the structure of ANFIS.

- Rule 1: If x is A_1_ and y is B_1_, then f_1_ = p_1_x + q_1_y+r_1_.
- Rule 2: If x is A_2_ and y is B_2_, then f_2_ = p_2_x + q_2_y+r_2_.

Where A1, A2 are fuzzy sets for the input parameter x and B1,B2 are fuzzy sets for input parameter y. Output of ANFIS model is denoted as f and the resultant parameters are denoted as p1, p2, q1, q2, r1 and r2.

#### Fuzzification layer

In this layer, each node produces a membership for a linguistic variable, and its output is calculated as follows:

3 $$\:{\text{O}}_{\text{i}}^{1}={{\upmu\:}}_{\text{A}\text{i}}\left(\text{x}\right)=\frac{1}{1+{\left[{\left(\frac{\text{x}-\text{v}\text{i}}{{\upsigma\:}\text{i}}\right)}^{2}\right]}^{\text{b}\text{i}}}$$.

Where x is represented as input value of node i and linguistic variable related with node i is A_i_. Function parameters are represented as σ_i_, v_i_ and b_i_.

#### Implication layer

Here neurons hold product of basic input parameters on the basis of weight. Computation of node output is formulated as follows.

4 $$\:{\text{O}}_{1}^{2} =w_i = \:{{\upmu\:}}_{\text{A}\text{i}}\left(\text{x}\right).{{\upmu\:}}_{\text{B}\text{i}}\left(\text{x}\right) \, \, \,\,\, \, i=1,2$$

Where w_i_ is represents the weight of neuron.

#### Normalization layer

In this layer by adding the weights of each neuron in this layer, neurons are fixed and normalised.

5 $$\:{O}_{1}^{3} =\:\stackrel{-}{{w}_{i}} = \:\frac{{w}_{i}}{\sum\:{w}_{i}}\, \, \,\,\, \, i=1,2$$

Where $\:\underset{\_}{{\text{w}}_{\text{i}}}$ represents neuron with normalized weight.

#### Defuzzification layer

In this Defuzzification Layer every neuron acts as an adaptive node by containing the consequent parameters of the system. Node output computation is formulated as follows.

6 $$\:{O}_{1}^{4} = \:\stackrel{-}{{w}_{i}} f_i = \:\stackrel{-}{{w}_{i}}(p_{i}x+q_{i}y+r_{i}) i=1,2$$

Where $\:\underset{\_}{{\text{w}}_{\text{i}}}$v the result of layer 3 and consequent parameters is are denoted as p_i_, q_i_ and r_i_.

#### Summation layer

In this layer, output is presented by single neuron by adding all the inputs. It is formulated as follows

7 $$\:{O}_{1}^{5} = f(x, y)= \:\sum\:\stackrel{-}{{w}_{i}}\:{f}_{i} = \:\frac{\varSigma\:iwifi}{\varSigma\:iwi} i=1,2$$

This ANFIS employs a hybrid learning method in which parameters are modified over the course of two passes using two separate optimization algorithms. The consequent parameters are modified during the forward pass when the inputs are given to ANFIS and the hypothesis parameters are maintained constant utilizing Least Square Estimation (LSE). The consequent parameters are refreshed in Layer 4 and the final output is computed as a result.

The backward pass begins once the final output is computed, and during this time the premise parameters are modified and the error is conveyed back to Layer 1. The ensuing parameters are fixed for this pass.

### Datasets

For validating the proposed scheme two datasets are utilized which are UNSW-NB15 dataset^21^ and CICDDoS2019 dataset^22^.

#### UNSW-NB15 dataset

The UNSW-NB 15 dataset’s raw network packets were generated by the IXIA PerfectStorm tool in the Cyber Range Lab of UNSW Canberra to produce a blend of real, existing normal activities and synthetic, current attack behaviours. 100 GB of the raw traffic were captured using the tcpdump utility. UNSW-NB15 dataset has set Nine distinct attacks are included in it, including DoS, malware, backdoors, and fuzzers. Raw network packets are part of the dataset. The testing set has 82,332 records from the several kinds, including attack and normal records, while the training set contains 175,341 records.

#### CICDDoS2019 dataset

This dataset provides a broad range of distributed denial of service attacks, the majority of which use amplification via reflection. The dataset, together with the CIC NIDS datasets IDS2017, IDS2018, and DoS2017, share a common set of features. 12,794,627 DDoS attack traces were collected as part of the CICDoS2019 data collection. Flow length, total forward packets, total reverse packets, and other features make up the 86 features. Benign, UDP, NETBIOS, UDP-LAG, NTP, LDAP, TFTP, SSDP, and MSSQL classifications are given to the dataset.

**Fig. 3:**
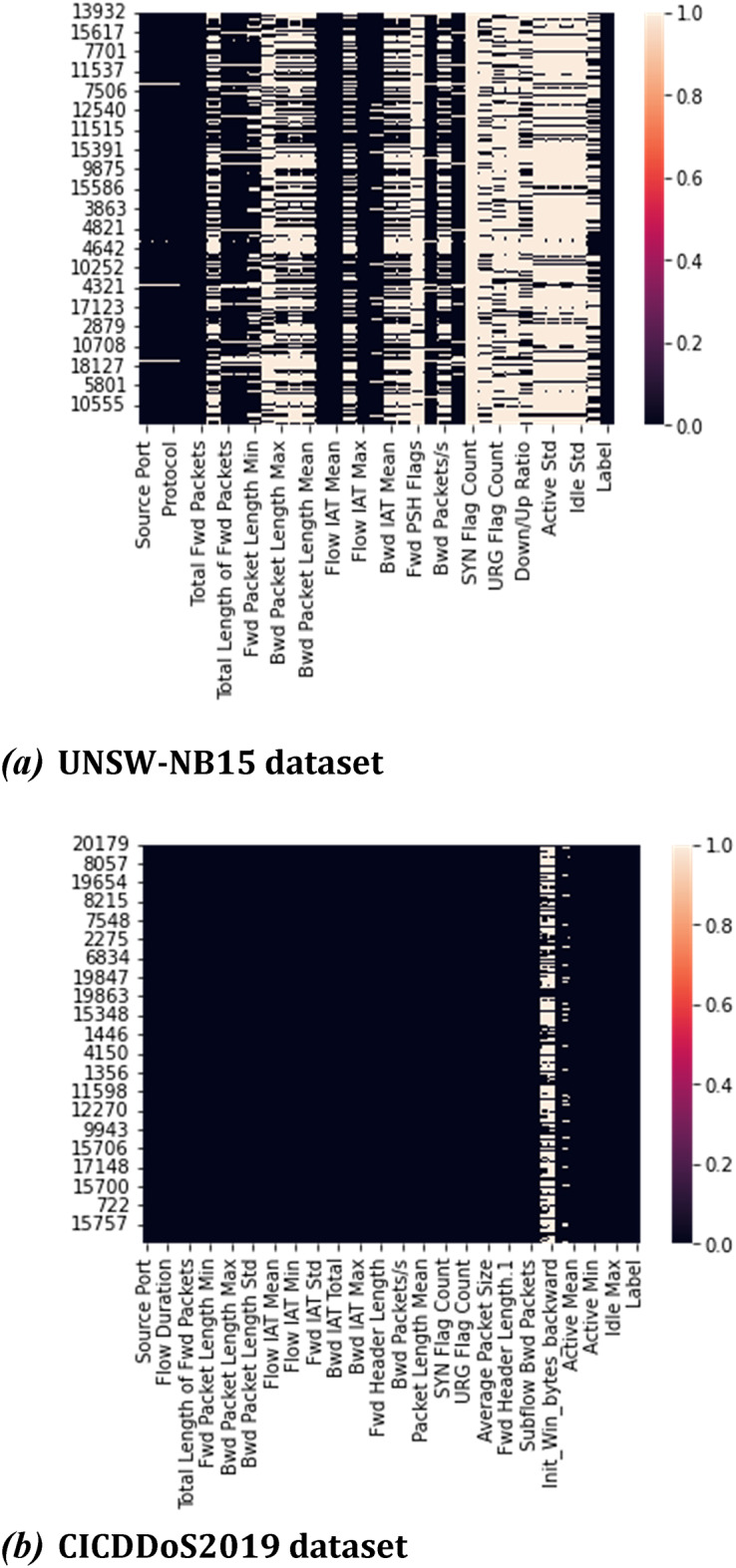
Heapmap projected for the major significant features.

#### Dataset preparation

- Figure 3 shows the heatmap projection and important significant feature selection for both the datasets.
- Gathering data values from the mentioned dataset of traffic information from an Intelligent Transport System (ITS), including traffic flow, speed, and occupancy data.
- Simulate a DDoS attack by generating a high volume of artificial traffic to the ITS using LOCUST traffic generation tool.
- Superimposing the simulated DDoS traffic into the dataset by merging it with the real traffic data.

The UNSW-NB15 and CICDDoS2019 datasets were selected due to their strong alignment with vehicular and denial-of-service attack scenarios in intelligent transportation systems. UNSW-NB15 contains diverse, real-world attack types mixed with normal traffic, making it effective for training general-purpose intrusion detection systems. CICDDoS2019 specifically focuses on distributed denial-of-service patterns across multiple attack vectors, which are highly representative of threats in ITS environments. Although newer datasets like CIC IoT 2023 offer updated attack types in broader IoT settings, our objective was to evaluate DDoS detection schemes in the vehicular context, where these benchmark datasets are still widely used and validated. As part of our future work, we plan to extend the proposed model to CIC IoT 2023 to assess generalizability across modern IoT-based ITS scenarios.

## Results

### Attack simulation

To simulate a DDoS attack in a ITS, an attacker can flood the network with a large number of packets, causing the network to become congested and causing delays in communication between the vehicles. The attacker can also target specific vehicles by sending packets to the vehicle’s MAC address, causing the vehicle to become overloaded and unable to respond to legitimate communication requests.

To simulate the attack, a traffic generator tool LOCUST is used to generate traffic in the network. The traffic generator is configured to generate traffic that simulates a DDoS attack by flooding the network with a large number of packets. The packets is crafted to target specific vehicles or to flood the network indiscriminately.

Once the attack traffic is generated, it is injected into the network using a network emulator. The network emulator tools such as CORE and Netkit are used to create a virtual ITS environment that simulates the behavior of the real network. The emulator is configured to replicate the network topology, the communication protocols, and the traffic patterns of the real network.

The experimental setup includes on-premises DDoS detection system which is deployed on the network that is designed to detect and mitigate DDoS attacks in the ITS. The system is deployed with certain rule-based methods to work along with the pfSense firewall that analyze the network traffic in real-time and identify patterns of malicious behavior.

The experimental evaluation that utilizes the CICDDoS2019 dataset for experimentation. The dataset has a collection of network traffic data that includes both normal and malicious traffic. The system is trained on both normal and malicious traffic.

The goal of the experimentation is to evaluate the performance of the proposed system in detecting and identifying malicious attacks in the network traffic.

**Fig. 4:**
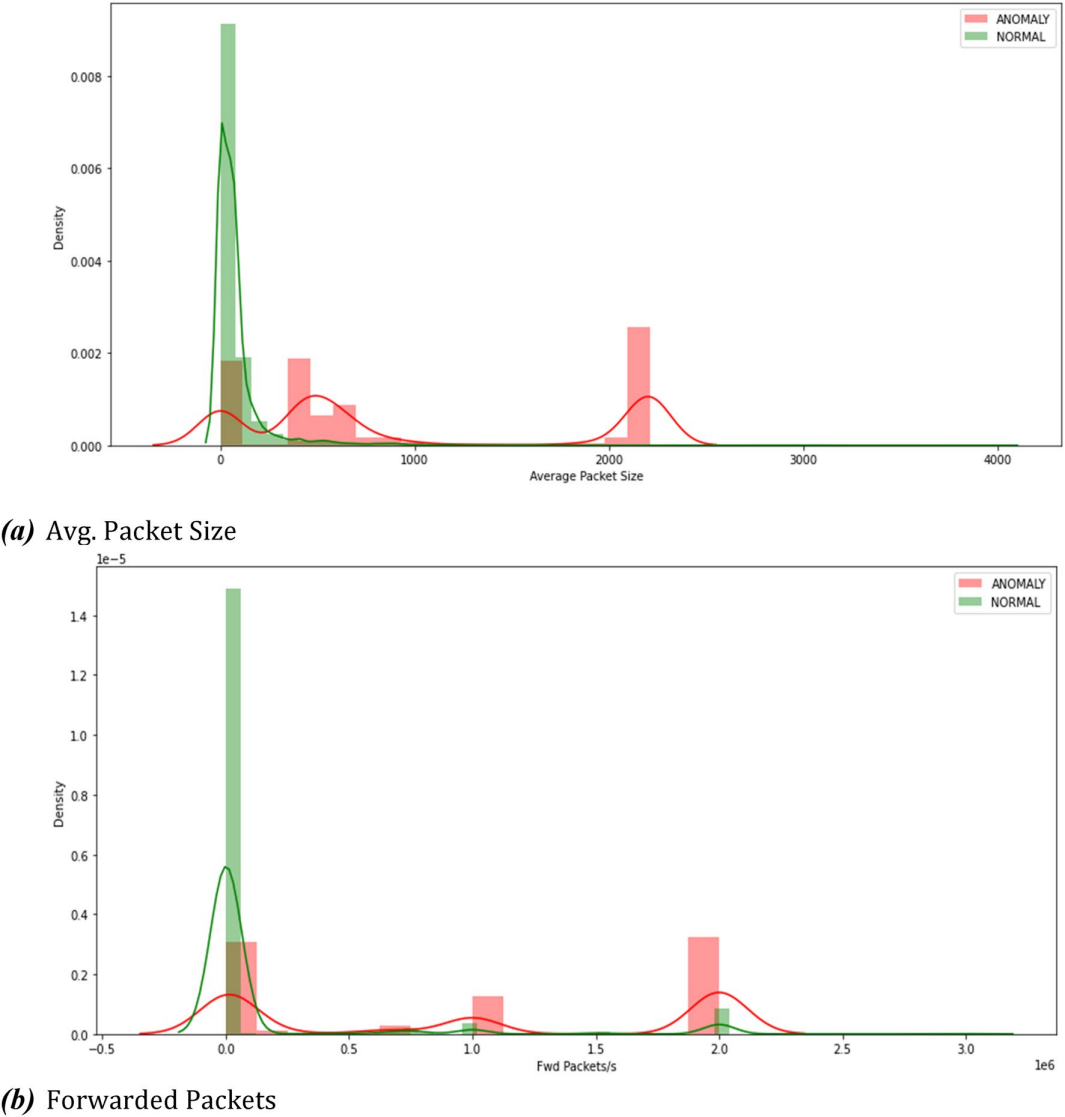
Traffic classification (anomalous traffic or Benign).

Figure 4 displays the overall statistics of the total number of attacks and differentiates the traffic into anomalous or benign. The traffic abnormality classification includes all the data packets including forwarded packets between other entities such as Road Side Units and other vehicles. This provides a deeper insight in breaking down the types of traffic that were used in the attacks and the total number of attacks that were carried out. This information can be useful for identifying patterns and trends in the attacks and for determining the most common types of files that are targeted by attackers.

## Discussion

**Fig. 5:**
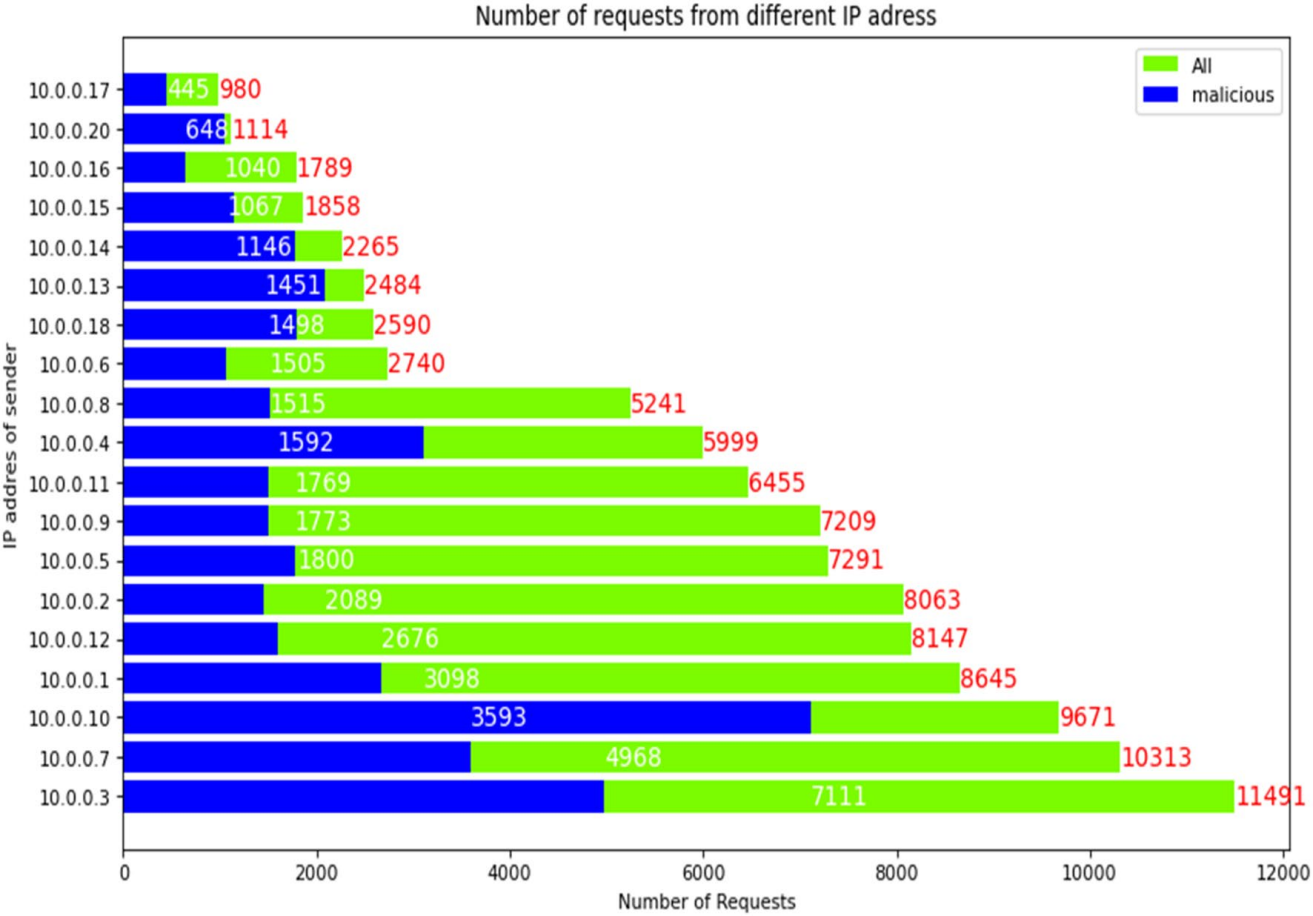
The overall statistics of the total attack and its requests.

Figure 5 shows the overall statistics of the total attack surface. The attack surface refers to the potential avenues of attack that are available to an attacker. This figure also provides an overview of the different attack surfaces that were targeted by the attackers (Different assets (IP)), which can help in identifying the potential vulnerable systems that need to be addressed to improve the security.

The experimental evaluation of the system concludes that all the attacks performed by the attacker seem to be malicious. This suggests that the proposed system was successful in detecting and identifying all the malicious attacks in the dataset. This is a positive result that demonstrates the effectiveness of the system in detecting and mitigating attacks in an Intelligent Transport System.

Overall, the statistics provided in the figures help in identifying patterns and trends in the attacks, which can be used to improve the security of the system. The experimental evaluation results demonstrate the effectiveness of the proposed system in detecting and identifying malicious attacks.

### Performance evaluation

To evaluate the performance of the system, the attack traffic is injected into the network and the system is monitored for its ability to detect and mitigate the attack. The performance of the system is evaluated using performance metrics such as accuracy, precision, recall, and F1-score. The system can also be evaluated for its ability to handle different types of DDoS attacks and for its scalability in handling large-scale attacks.

The effectiveness of the proposed ANFIS-based DDoS detection system was evaluated using the UNSW-NB15 and CICDDoS2019 datasets. To validate the model, we conducted experiments comparing ANFIS with traditional classifiers including Support Vector Machines (SVM), Random Forest (RF), XGBoost, and CNN.

### Experimental setup

Datasets Used: UNSW-NB15, CICDDoS2019.

Train-Test Split: 80% training, 20% testing.

Evaluation Metrics: Accuracy, Precision, Recall, F1-score, False Positive Rate (FPR).

Hardware: Intel i7 12th Gen CPU, 16GB RAM, NVIDIA RTX 3060 GPU.

Software: Python 3.9, TensorFlow, Scikit-Learn.

### Evaluation metrics

Throughout the evaluation phase, a variety of evaluation metrics are employed to gauge the effectiveness of the scheme and identify its positive and negative aspects. This is done in order to understand the scheme’s operation better. It is necessary to analyse a model in order to decide whether it can be used in the beginning stages of the study.

### Accuracy

By comparing the percentage of correctly categorised DDoS attacks and common occurrences to the overall number of occurrences, the suggested detection scheme’s accuracy is demonstrated.

$$\:\text{A}\text{c}\text{c}\text{u}\text{r}\text{a}\text{c}\text{y}=\frac{\text{T}\text{P}+\text{T}\text{N}}{\text{T}\text{P}+\text{T}\text{N}+\text{F}\text{P}+\text{F}\text{N}}$$

**Fig. 6:**
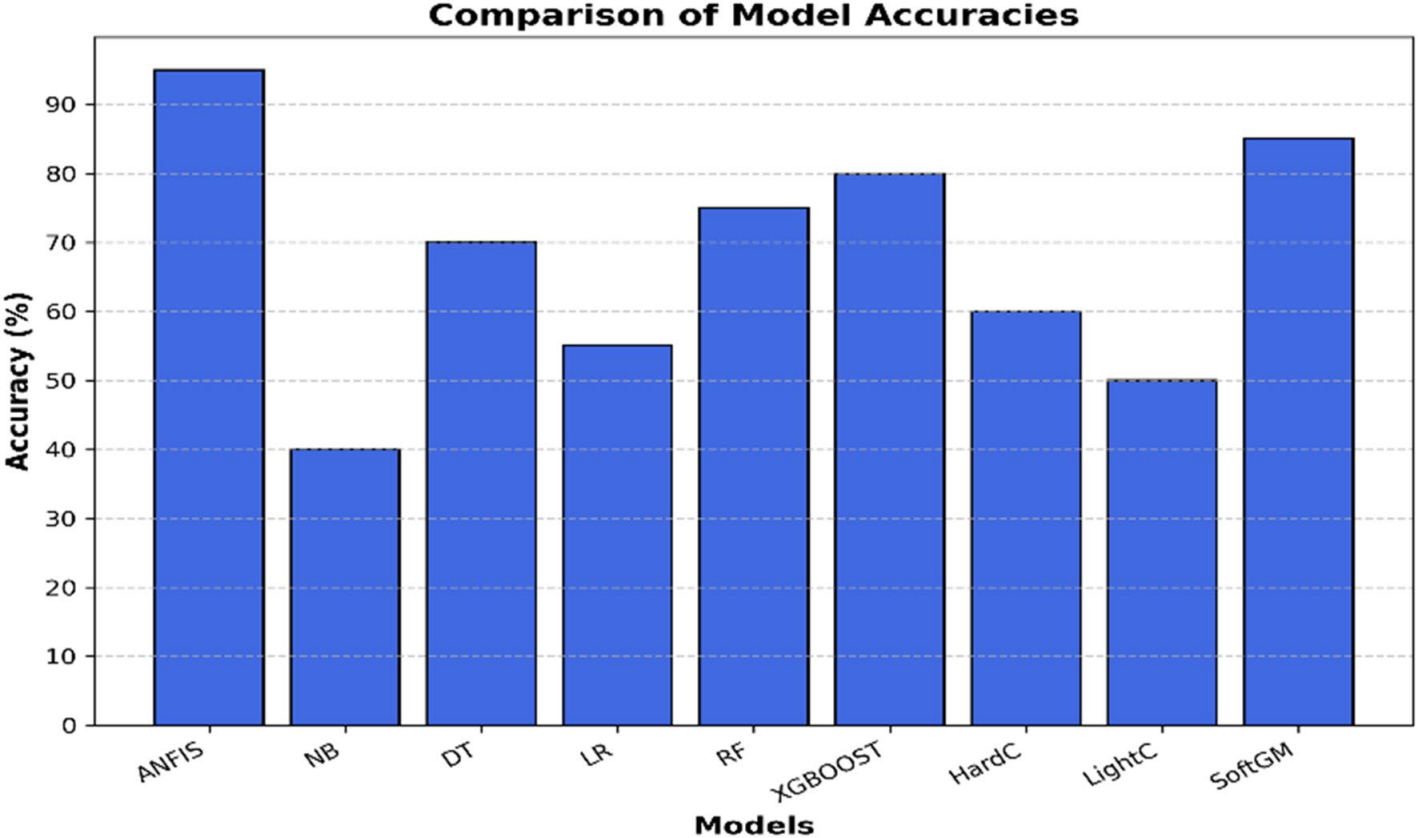
The performance analysis of the proposed solution vs. the state of the art classifiers.

True Positive (TP) stands for an attack incident that has been accurately categorised, whereas True Negative (TN) stands for an normal occurrence. False Positive (FP) suggests improper classification of normal instances, while False Negative (FN) implies incorrect classification of assault incidents. Figure 6 shows the accuracy of the proposed solution over the superimposed dataset and its parameters.

### Precision

The efficiency of the proposed framework is predicted by estimating the fraction of attack observation.

$$\:\text{P}\text{r}\text{e}\text{c}\text{i}\text{s}\text{i}\text{o}\text{n}=\frac{\text{T}\text{P}}{\text{T}\text{P}+\text{F}\text{P}}$$

### Recall

It displays real-time attack categorization in percentage form and uses that information to compute the overall rate of success of the suggested scheme.

$$\:\text{R}\text{e}\text{c}\text{a}\text{l}\text{l}=\frac{\text{T}\text{P}}{(\text{T}\text{P}+\text{F}\text{N})}$$

### F1 score

A symmetrical average of the precision and recall measures is used to determine the F1 score. To improve forecasting accuracy, it performs as a statistical function.

$$\:\text{F}1\:\text{s}\text{c}\text{o}\text{r}\text{e}=\frac{2\text{*}\text{T}\text{P}}{2\text{*}\text{T}\text{P}+\text{F}\text{P}+\text{F}\text{N}}$$

The comparative performance metrics of the proposed ANFIS model and other baseline classifiers are presented in Table 2.

**Table 2:** Comparative performance analysis.

Model	Accuracy (%)	Precision (%)	Recall (%)	F1-score (%)	False positive rate (%)
Support vector machine	86.3	84.5	85.1	84.8	7.9
Random forest	89.7	88.9	89.4	89.1	6.3
XGBoost	91.8	91.0	90.8	90.9	5.8
Convolutional neural network	93.5	93.0	92.7	92.8	5.2
**ANFIS (proposed)**	**94.3**	**94.1**	**93.8**	**93.9**	**4.8**

As shown in Table 2, the ANFIS model consistently outperforms other models across all metrics, justifying its suitability for ITS-based DDoS detection.

The ANFIS-based model achieved the highest accuracy (94.3%), outperforming traditional machine learning models such as SVM (86.3%), Random Forest (89.7%), and XGBoost (91.8%). While CNN achieved close performance (93.5%), ANFIS had a lower false positive rate (4.8%), making it more reliable for real-time detection.

While this study compares the proposed ANFIS model with widely adopted ML and DL baselines (SVM, RF, CNN, XGBoost), the selection reflects a focus on interpretability, computational feasibility, and reproducibility in real-time ITS environments. Although hybrid deep learning and optimization-based models (e.g., CNN-GA, LSTM-PSO) have shown high accuracy, they often introduce significant complexity and latency, which can be challenging in resource-constrained ITS deployments. Incorporating such models remains an important future direction for extending this study’s benchmarking scope.

In the context of ITS, even small false positive rates triggering unnecessary alerts, affect routing systems, or disrupt vehicle coordination. Based on related ITS literature, FPRs below 5% are considered acceptable for operational safety, provided the detection system is lightweight and responsive. Our model’s FPR of 4.8% meets this criterion. However, further refinement using real-time feedback mechanisms or adaptive learning is essential for long-term deployment.

The superior performance of ANFIS can be attributed to:

Hybrid Learning Mechanism: The combination of fuzzy logic and neural networks enables better adaptability to dynamic traffic conditions.

Lower False Positives: The use of fuzzy membership functions improves classification granularity, reducing misclassification of normal traffic as attacks.

Computational Efficiency: Compared to deep learning models like CNN, ANFIS requires less computational power, making it suitable for real-time ITS applications.

While ANFIS performed well in most cases, its training time was slightly higher than XGBoost due to rule-based processing.

The model could further benefit from hybrid optimization techniques (e.g., genetic algorithms, swarm intelligence) to fine-tune fuzzy rules dynamically.

Practical Implications:

The proposed ANFIS-based detection model is lightweight, interpretable, and requires minimal computational resources, making it suitable for real-time deployment in Intelligent Transportation Systems. It can be integrated with roadside infrastructure, edge gateways, or vehicular units to monitor network activity and detect DDoS attacks with minimal delay. The low false positive rate reduces unnecessary alerts, supporting stable traffic communication and improved road safety in smart transportation ecosystems.

Limitations:

The proposed ANFIS model, while achieving high accuracy and low false positives, has certain limitations. Its rule-based structure can become complex with large feature sets or multi-class scenarios, and training time increases with the number of fuzzy rules. The model’s effectiveness may reduce with previously unseen attacks, requiring periodic updates or adaptive mechanisms.

While the feature set was intentionally constrained to manage rule complexity, we acknowledge that scaling to denser traffic or sensor environments may introduce challenges. The fuzzy rule explosion problem and increased training time could affect real-time efficiency in large-scale urban ITS scenarios. Future work will involve evaluating the model’s behavior under high-volume traffic and exploring hierarchical or modular extensions to preserve performance.

Although LOCUST-based simulations emulate high-throughput DDoS behavior in real-time, they do not fully capture real-world vehicular dynamics like mobility, wireless delays, or edge constraints. Future work will address this through deployment in an operational ITS testbed.

The evaluation, based on UNSW-NB15 and CICDDoS2019, covers diverse DDoS patterns but may not reflect all traffic contexts, such as urban vs. rural deployments or rare attacks. The ANFIS architecture, however, supports adaptability and will be extended with synthetic scenarios and additional datasets to improve generalizability.

The feature selection in this work was based on entropy and information gain due to their interpretability and computational efficiency in real-time scenarios. However, we acknowledge that such filter-based approaches may overlook complex non-linear feature interactions. Recent strategies using deep learning models, SHAP analysis, or autoencoder-based feature selection can capture higher-order dependencies. Future work will explore these advanced techniques to reduce selection bias and further improve detection robustness.

Finally, while membership functions are adaptively tuned, the fuzzy rules were manually defined. Future work will incorporate rule evolution techniques to better align with evolving cyber threat landscapes.

## Conclusion

In the interests of addressing the security goals of the intelligent transport system, a DDoS attack detection scheme based on Adaptive Neuro-Inference System (ANFIS) is proposed in this work. ANFIS-based DDoS detection in ITS is a powerful technique that can effectively detect DDoS attacks in real-time. Its ability to adapt to changing network traffic patterns and its high accuracy make it a promising approach for network security. However, it is crucial to combine ANFIS with other detection techniques to create a robust and comprehensive DDoS detection system. The shortcomings of the currently presented methods are taken into account, and our new detection scheme will get around them. The security flaws need to be fixed because of the high dynamic nature of the V2V and V2I vehicular communication. In ANFIS, the fuzzy logic model and artificial neural networks’ (ANN’s) learning strategy are combined. Performance of the proposed scheme is evaluated by considering the metrics like accuracy, precision, recall, and F1 score. The proposed system produced better outcomes according to the experiment’s findings. Future research can focus on extending the model for multi-class intrusion detection and evaluating its performance on recent datasets such as CIC IoT 2023. Additionally, integrating metaheuristic optimization techniques into dynamically fine-tune fuzzy rules and membership functions may further improve detection accuracy and adaptability in evolving ITS environments.

## Electronic supplementary material

Below is the link to the electronic supplementary material.

Supplementary Material 1

## Data Availability

The datasets generated and analysed during the current study are available in the CICDDoS2019, UNSW-NB15 repository, WEB LINK TO DATASETS: https://www.kaggle.com/datasets/rodrigorosasilva/cic-ddos2019-30gb-full-dataset-csv-files, https://www.kaggle.com/datasets/mrwellsdavid/unsw-nb15.
